# LncTUG1 promotes hepatocellular carcinoma immune evasion via upregulating PD-L1 expression

**DOI:** 10.1038/s41598-023-42948-8

**Published:** 2023-10-09

**Authors:** Rongshou Wu, Weiwei Liu, Qingping Yang, Jingling Zhang, Ping Hou, Jianghui Xiong, Linquan Wu, Enliang Li

**Affiliations:** 1 Department of Hepatobiliary and Pancreatic Surgery, The Second Affiliated Hospital of Nanchang University, Nanchang, 330006 Jiangxi People’s Republic of China; 2 Department of Hepatobiliary Surgery, Xinqiao Hospital, Third Military Medical University, 83 Xinqiao Main Street, Chongqing, 400000 People’s Republic of China; 3 Department of Assisted Reproductive, The First Affiliated Hospital of Nanchang University, Nanchang, 330006 Jiangxi People’s Republic of China; 4 Department of Anesthesiology, The First Affiliated Hospital of Nanchang University, Nanchang, 330006 Jiangxi People’s Republic of China

**Keywords:** Cancer, Cancer microenvironment, Cancer therapy, Tumour immunology

## Abstract

HCC is one of the most common malignant tumors worldwide. Although traditional treatment methods have been improved in recent years, the survival rate of HCC patients has not been significantly improved. Immunotherapy has shown extremely high clinical value in a variety of tumors. In this study, we found that TUG1 could regulate the expression of PD-L1 through JAK2/STAT3 to mediate immunosuppression. Here, The expression of TUG1 and PD-L1 in HCC tissues was evaluated through analysis of databases and verified in HCC tissue and HCC cancer cells by qRT-PCR. The effect of TUG1 on tumor immune escape was detected by coculture, and cell viability was detected with a CCK8 assay. The results demonstrated that TUG1 was closely associated with anticancer immunity. TUG1 and PD-L1 were highly expressed in HCC tissues and HCC cancer cells, and high expression of TUG1 and PD-L1 was related to the poor prognosis of HCC patients. In addition, knocking down TUG1 expression could reduce PD-L1 expression and enhance the cancer cell-killing capability of T cells. Downregulating TUG1 expression could also decrease the mRNA and protein expression of JAK2 and STAT3. To sum up, TUG1 and PD-L1 are overexpressed in patients with liver cancer and are related to the poor prognosis of these patients. Silencing TUG1 expression reduced the mRNA and protein expression of PD-L1 by affecting the JAK2/STAT3 pathway.

## Introduction

Hepatocellular carcinoma (HCC) is one of the most common malignant tumors worldwide and one of the tumors with the highest mortality rate^1^. Currently, therapies of HCC include hepatectomy, liver transplantation and tumor ablation, as well as other palliative treatments such as transarterial chemoembolization (TACE) including conventional TACE and Drug-eluting bead (DEB)-TACE and sorafenib^2,3^. Although some progress has been made in the treatment of liver cancer, the prognosis of HCC is not ideal because HCC has the characteristics of metastasis and high recurrence^1^^,^^4^. These observations indicate the urgent need to find new diagnostic and treatment options.

Tumor immune escape plays an extremely important role in the progression of tumors. Programmed cell death receptor-1 (PD-1) and programmed cell death ligand (PD-L1) have been shown to play irreplaceable roles in tumor immune escape^5,6^, are widely expressed on the surface of a variety of cells and are considered costimulatory molecules related to negative immune regulation^7,8^. It has been reported that PD-1/PD-L1 is very likely involved in malignant tumor progression and lymph node metastasis^9–11^. Fortunately, in recent clinical trials, it has also been shown that PD-1 inhibitors have significant therapeutic effects on adult patients with advanced and/or refractory solid tumors^12,13^. Therefore, furthering the understanding of the regulatory mechanism of PD-L1 in tumors could help improve immunotherapy.

Taurine upregulated gene (TUG1) is identified as a transcript whose expression is upregulated by taurine treatment^14^. The role of LncTUG1 has been reported in various cancers, such as triple-negative breast cancer^15^, osteosarcoma^16^, acute myeloid leukemia^17^, and hepatocellular carcinoma^18^. These studies have mainly focused on the correlation of LncTUG1 with tumor progression, but whether lncTUG1 affects antitumor immunity has been reported in only a limited capacity. Tumor immune microenvironment (TIME) plays an important role in tumorigenesis and therapeutic response^19^. Tumor immune microenvironment is also an important factor in regulating immune escape. The infiltration of immune cells in the tumor microenvironment plays an important and complex role in the formation and development of tumors. Interestingly, research shows that the expression of TUG1 was positively correlated with the infiltration levels of immune cells in liver cancer tissues^20^. TUG1 has an important effect on tumor immune microenvironment of HCC. Therefore, we examined the mechanism by which TUG1 interacts with PD-L1 in HCC and evaluated its contribution to tumor immunology. Therefore, we may provide a beneficial direction to enhance the effect of PD-1/PD-L1-targeting treatments.

## Materials and methods

### Human tissue specimens

Hepatocellular carcinoma and adjacent normal tissue samples were collected from 36 diagnosed patients who received surgical treatment at the Second Affiliated Hospital of Nanchang University during 2018 and 2020. None of the patients received any medications for treatment before sample collection. All tissue samples were then immersed in liquid nitrogen and stored at − 80 °C for later use. All studies in this project were approved by the Ethics Committee of the Second Affiliated Hospital of Nanchang University. Prior to the collection of any samples, all participants signed an informed consent form, which complied with the Declaration of Helsinki.

### Cell culture and treatments

Human normal liver cells (HL-7702) and four HCC cell lines (SMMC7721, MHCC97H, HCCLM3 and Huh-7) were purchased from the Shanghai Institute of Cell Biology (Shanghai, China). All cell lines were cultured in high-glucose DMEM (Solarbio, Beijing, China) supplemented with 10% FBS (Bio Industries, Beit-Haemek, Israel), 100 µg/mL streptomycin and 100 U/mL penicillin at 37 °C and incubated in 5% carbon dioxide. To treat HCC lines, JAK2 inhibitor (10 µmol/ml, AZ960,Adooq Bioscience), STAT3 inhibitor (10 µmol/ml, A12232, Adooq Bioscience), and recombinant human IFN-γ (100 ng/mL; PeproTech) were added to the medium for 24 h.

### Analysis of publicly available data

We estimated TUG1 expression in various cancers, and TUG1 expression was correlated with patient prognosis and immune checkpoint genes in various cancers by TIMER^21^(http://timer.comp-genomics.org/). TIMER provides immune infiltrate abundances estimated by multiple immune deconvolution methods and allows users to dynamically generate high-quality Figs to comprehensively explore immunological, clinical and genomic features of tumors. We evaluated LncTUG1 as an anticancer immunity-related lncRNA with ImmLnc^22^. ImmLnc is a web-based resource for investigating the immune-related functions of lncRNAs across cancer types. In this resource, users can query lncRNA pathways, lncRNA-immune cell type correlations, and cancer-related lncRNAs across 33 cancer types. The prognosis of patients with TUG1 expression was confirmed by UALCAN analysis (http://ualcan.path.uab.edu/). UALCAN now provides long noncoding RNA (lncRNA) expression and patient survival information across cancer types using level 3 RNA-seq (hg38) TCGA datasets^23–25^. The correlation of TUG1 and PD-L1 expression was also confirmed by Gene Expression Profiling Interactive Analysis (GEPIA2) (http://gepia2.cancer-pku.cn/). Patient prognosis was related to TUG1 expression with The Cancer Proteome Atlas (TCPA) (https://www.tcpaportal.org/).

### Transfection

For stable silencing of TUG1 expression in HCC cell lines, nonsense and TUG1 shRNA plasmids were manufactured by Jikai Gene Biology Co., Ltd. (Shanghai, China). The sequences of shTUG1:GCTTGGCTTCTATTCTGAATCCT. JAK2 (NCBI Ref Seq: NM_004972.3) and STAT3 (NCBI Ref Seq:NM_139276.2) overexpression plasmids were manufactured by Sino Biological. Lipofectamine 3000 and P3000 (Invitrogen Life Technologies, Carlsbad, California) were used to transfect cells.

### Western Blot analysis

Standard Western blotting procedures were used to detect protein expression in tissues and cells, as described previously^26^. Briefly, tissue and cell proteins were extracted with RIPA buffer with 1% phenylmethanesulfonyl (PMSF); the extracted protein was separated by 10% SDS/PAGE and transferred to polyvinylidene fluoride membranes (Millipore, USA). Then, the membranes were incubated with a primary antibody overnight at 4 °C. The membranes were washed three times with 1 × TBST for 10 min each time. Subsequently, they were incubated with secondary antibodies at room temperature for 1 h. Finally, the blots were detected and analyzed with software (Bio-Rad, Hercules, California, USA). Protein detection was performed by using the following primary antibodies: anti-PD-L1 (ab205921, Abcam), anti-GAPDH (ab8245, Abcam), anti-JAK2 (#3230, Cell Signaling Technology), anti-pJAK2 Tyr1007/1008 (#3771,Cell Signaling Technology),anti-STAT3 (#9139, Cell Signaling Technology), anti-pSTAT3 Tyr705 (#9145, Cell Signaling Technology). Western blot experiments in the manuscript were performed by multiple authors. The C, D and E in Fig. 4 is the original unprocessed images. The blots of the C, D and E in Fig. 3 and supplementary Fig. 2B were cut prior to hybridisation with antibodies. So these parts of original images of full-length blots cannot be provided.

### Real-time quantitative PCR analysis

Total RNA was extracted from HCC cells or clinical tissues using TRIzol (Invitrogen, USA) according to the manufacturer’s instructions. Reverse transcription was performed to synthesize complementary DNA followed the instructions of a reverse transcription kit (TransGen Biotechnology Co., Ltd., Beijing, China). Next, reverse transcription quantitative polymerase chain reaction (RT-qPCR) was performed using an ABI PRISM 7500 system (ABI, Inc. Oyster Bay, New York, USA) according to the instruction kit of SYBR Premix Ex TaqTM II (Takara Biotechnology Co., Ltd., Dalian, China). Primers were synthesized and provided by the Beijing Institute of Genomics (China) (Supplemental Table 1). The values of target genes were calculated by using the 2^–ΔΔCt^ method. Glyceraldehyde-3 phosphate dehydrogenase (GAPDH) was used as an endogenous control. The 2^-∆∆CT^ method was employed to determine the relative mRNA expression in Human normal liver cells and four HCC cell lines cells, while the relative mRNA expression in hepatocellular carcinoma and adjacent normal tissue samples was calculated using 2^-∆CT^.

**Table 1:** Differential expression of lncTUG1 in various cancers.

Cancer	IncRNA symbol	*P* value	*P* adjust	FC ratio	Test type
HNSC	TUG1	0.000	0.000	1.471	t_test
KIRP	TUG1	0.000	0.000	1.205	t_test
LIHC	TUG1	0.000	0.000	1.423	t_test
LUAD	TUG1	0.000	0.000	1.243	t_test
STAD	TUG1	0.000	0.000	1.433	t_test
BLCA	TUG1	0.002	0.007	1.303	t_test
ESCA	TUG1	0.001	0.007	1.387	t_test
PRAD	TUG1	0.025	0.047	1.072	t_test
KIRC	TUG1	0.103	0.13	1.067	t_test

*P* Value: The *p*-values for GSEA analysis; *P* Adjust: The adjusted *p*-values; FC Ratio: The fold changes or odd ratios; Test Type: The methods used for identifying the lncRNA.

### T cell-mediated tumor cell killing assay

T cells were suspended in ImmunoCult™-XF Expansion Medium (STEMCELL, catalog #10981) and activated with an anti-CD3/CD28 T cell activator (STEMCELL, catalog #10917). After 3 days of directly coculturing tumor cells and T cells in 24-well plates, the wells were washed with PBS three times to remove the T cells, and tumor cell viability was measured with a CCK-8 assay.

### Statistical analysis

Statistical analyses were performed using GraphPad Software (version 7.0, CA, USA). Data for continuous variables are expressed as the mean ± standard deviation (SD) and were analyzed using Student’s t-test^24^; *P* < 0.05 was considered significant.

## Results

### LncTUG1 is identified as an immune-related lncRNA in HCC

**Figure 1:**
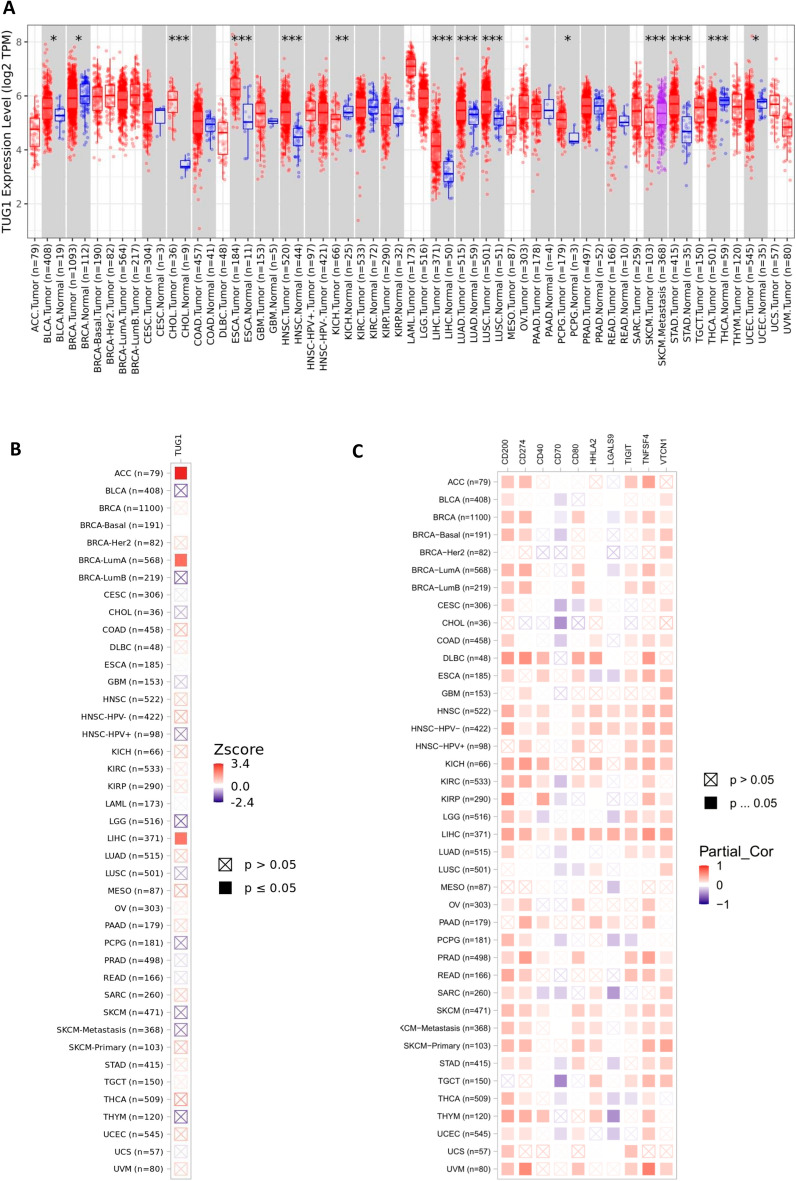
Analysis of TUG1 expression in various cancer types by TIMER. (**A**): Deferential mRNA expression of TUG1 between tumor and normal tissue samples for various cancers. (**B**) Heat map showing the association between TUG1 mRNA expression and clinical outcomes in various cancers. (**C**) Heat map showing the correlation between TUG1 and some immune checkpoint molecules in various cancer types.

We first analyzed TUG1 expression in various cancers and found that TUG1 was especially highly expressed in CHOL, CESC, HNSC, LIHC, LUAD, LUSC, and STAD tumor tissues (Fig. 1A). Moreover, we found that high TUG1 expression significantly impacted the prognosis of ACC, BRCA-LumA, and LIHC patients (Fig. 1B). ImmLnc online analysis results also indicated that TUG1 expression was perturbed in various cancers, including HNSC, KIRP, LIHC, LUAD, STAD, BLCA, and ESCA (Table 1). In addition, to further explore the relation of LncTUG1 expression with immunity in cancer, we assessed the correlation of TUG1 expression with that of some immune checkpoint molecules (CD200, CD274, CD40, CD70, CD80, HHLA2, LGALS9, TIGIT, TNFSF4, and VTCN1) in various cancers (Fig. 1C).

The expression of TUG1 was significantly positively correlated with that of immune checkpoint molecules in HCC, including TNFSF4, CD200, and CD274. We also found that the expression of TUG1 was correlated with immune cell infiltration in HCC, such as that of CD4 T cells and macrophages (Table 2). In hepatocellular carcinoma, TUG1 was found to play important roles in immune-related pathways, such as antigen processing and presentation, and chemokine receptors (Table 3). These results imply that LncTUG1 may play critical roles in anti-HCC immunity.

**Table 2:** Correlations of LncTUG1 with immune-related pathways in HCC.

Immune pathway	*P* value	*P* adjust	ES	Score
Antigen processing and presentation	0.002618	0.044503	− 0.54812	− 0.99476
Chemokine receptors	0.015025	0.127713	0.541226	0.96995
Antimicrobials	0.043636	0.150609	− 0.29187	− 0.91273
TCR signaling pathway	0.053156	0.150609	0.416174	0.893688
TGFb family member	0.048193	0.150609	0.568991	0.903614
TNF family members	0.027076	0.150609	0.705388	0.945848
TGFb family member receptor	0.099278	0.241104	0.64461	0.801444
Interleukins	0.36342	0.772268	− 0.41121	− 0.27316
BCR signaling pathway	0.416804	0.787296	0.327376	0.166392
Cytokine receptors	0.490991	0.834685	0.269297	0.018018
Cytokines	0.606775	0.91498	0.255899	− 0.21355
Interleukins receptor	0.645868	0.91498	0.32067	− 0.29174
Chemokines	0.971808	0.98265	0.225904	− 0.94362
Interferons	0.871227	0.98265	− 0.55881	0.742455
Interferon receptor	0.913127	0.98265	0.405205	− 0.82626
Natural killer cell cytotoxicity	0.98265	0.98265	0.215606	− 0.9653
TNF family members receptors	0.970639	0.98265	0.233	− 0.94128

Immune Pathway: The immune-related pathways; *P* Value: The *p*-values for GSEA analysis; *P* Adjust: The adjusted *p*-values; ES: Enrichment scores; Score: The lncRES scores for lncRNA-immune pathway pairs; Marker Gene Number: The number of marker genes in specific pathway.

**Table 3:** Correlations of LncTUG1 expression and immune cell infiltration levels in cancer.

IncRNA symbol	Immune cell	*P* value	Rs value
TUG1	CD8 T cell	0.724	− 0.018
TUG1	Dendritic	0.444	0.04
TUG1	B cell	0.047	0.103
TUG1	Neutrophil	0.041	0.106
TUG1	Macrophage	0.000	0.181
TUG1	CD4 T cell	0.000	0.241

Immune Cell: The immune cell types; *P* Value: The *p*-values for correlation coefficient; R Value: The correlation coefficient.

### TUG1 and PD-L1 are highly expressed and positively correlated in HCC tissues and cells

**Figure 2:**
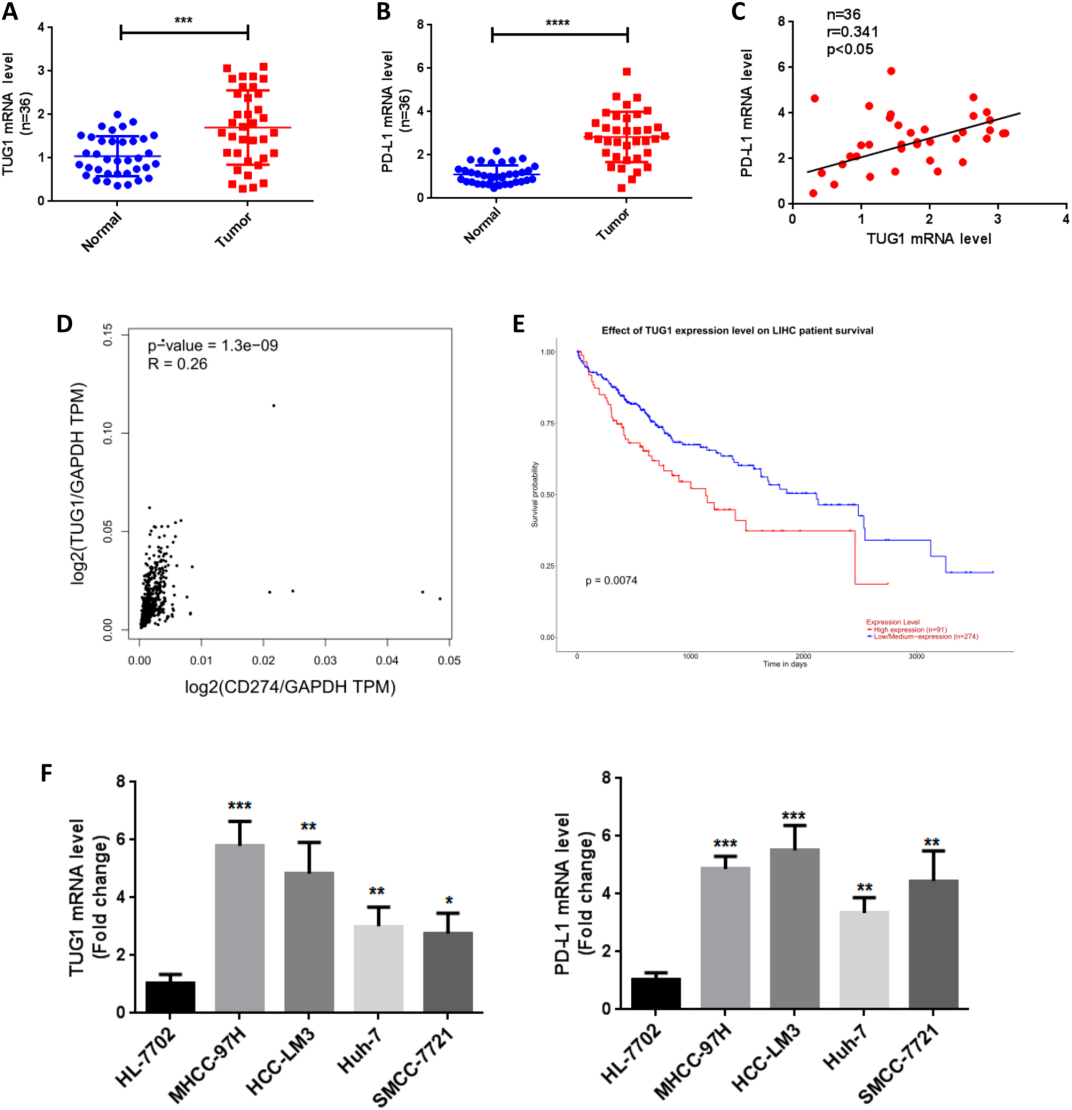
The expression of TUG1 and PD-L1 is increased and indicates a poor prognosis in HCC. (**A**): The relative mRNA expression of TUG1 in 36 paired HCC tumor and adjacent normal tissue samples was measured by qRT-PCR. (**B**): The relative mRNA expression of PD-L1 in 36 paired HCC tumor and adjacent normal tissue samples was measured by qRT-PCR. (**C**): The correlation between TUG1 and PD-L1 expression in HCC tissue samples was determined. (**D**): The correlation between TUG1 and PD-L1 expression in HCC tissue samples was determined by analysis with GEPIA2. (**E**): The prognosis of HCC patients according to TUG1 expression was evaluated by TCPA analysis. (**F**): The relative mRNA expression levels of TUG1 and PD-L1 in a normal liver cell line (HL-7702) and liver cancer cell lines (HCC-LM3, MHCC-97H, Huh-7, SMCC-7721) were measured by qRT-PCR. *p < 0.05, **p < 0.01, ***p < 0.001, ****p < 0.0001.

Accumulating studies indicate that lncRNAs can sponge various miRNAs, which subsequently affects PD-L1 expression^27^. Therefore, we further analyzed the potential of the interaction between LncTUG1 and PD-L1. We first collected a total of 36 human HCC tumor and adjacent normal tissue samples. TUG1 and PD-L1 expression was markedly increased in the tumor samples compared to the paired normal tissue samples by qRT-PCR analysis (Fig. 2A,B). Of note, the expression of TUG1 was positively correlated with PD-L1 expression in HCC tissues (Fig. 2C), which was confirmed with data from The Cancer Genome Atlas (TCGA) and GTEx databases by analysis with GEPIA2 (Fig. 2D). We also found that high TUG1 expression was associated with a poor prognosis in HCC by analysis of TCGA data (Fig. 2E). In addition, we examined TUG1 and PD-L1 expression in HL-7702 normal human hepatic cells and common HCC cell lines, including MHCC-97H, HCC-LM3, Huh-7, and SMCC-7721. The RNA expression levels of TUG1 and PD-L1 were significantly higher in the HCC cell lines than in HL-7702 cells (Fig. 2F). Because TUG1 and PD-L1 expression was higher in the MHCC-97H and HCC-LM3 cell lines than in the Huh-7 and SMCC-7721 cell lines, we selected the MHCC-97H and HCC-LM3 HCC cell lines for further study.

### Downregulation of TUG1 expression inhibits the expression of PD-L1 and enhances antitumor immunity in vitro

**Figure 3:**
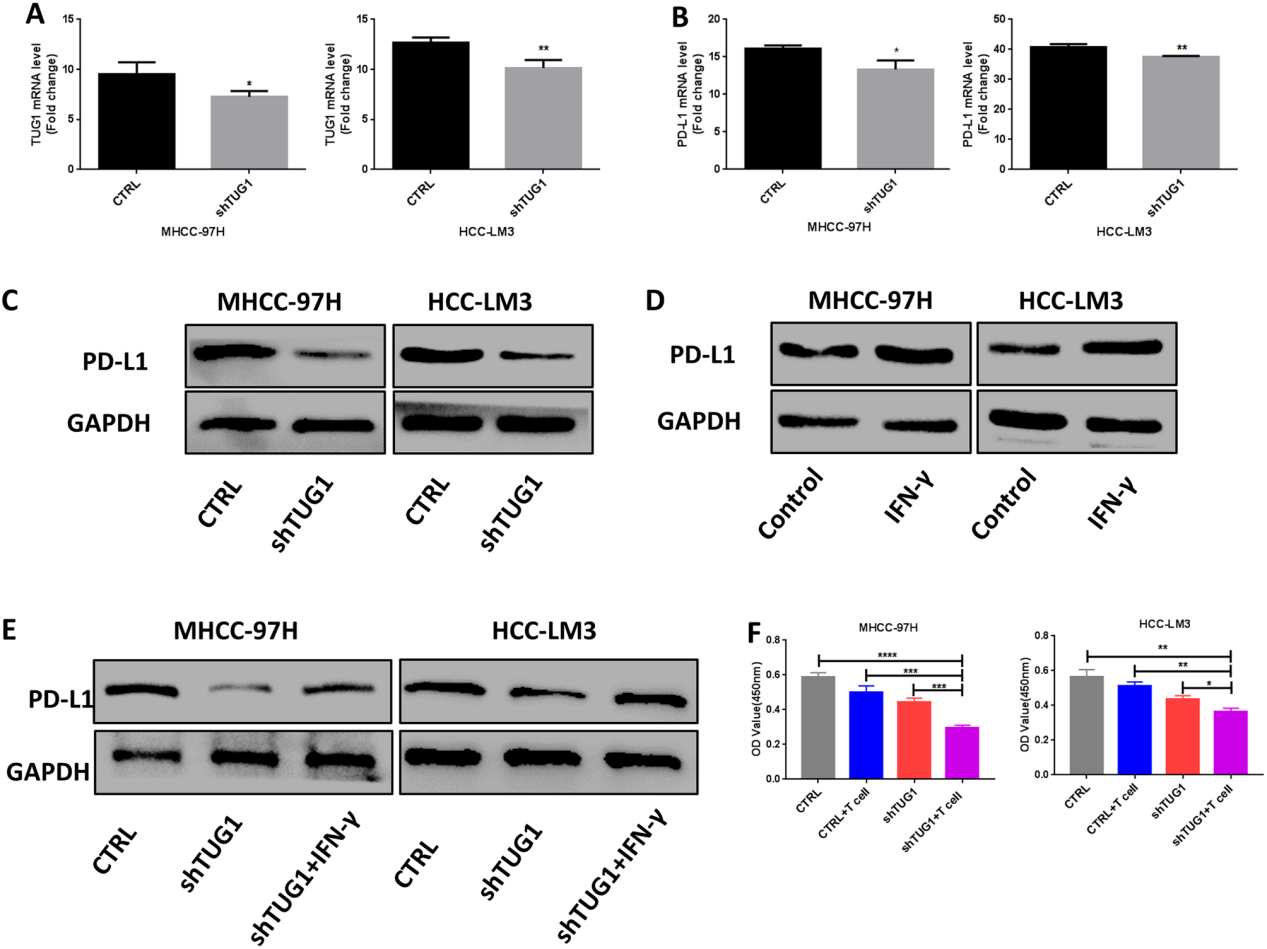
Silencing TUG1 expression reduced PD-L1 expression. (**A**): Relative TUG1 expression was determined by qRT-PCR. (**B**): The relative mRNA expression of PD-L1 was reduced in shTUG1 HCC cells. (**C**): The total PD-L1 protein level was reduced in shTUG1 HCC cells. (**D**): The PD-L1 protein level was upregulated under IFN‐γ stimulation. (**E**): IFN-γ stimulation restored PD-L1 protein expression in shTUG1 HCC cells. (**F**): Silencing TUG1 expression in HCC cells improved T cell-mediated killing in vitro. To treat HCC lines, recombinant human IFN-γ (100 ng/mL; PeproTech) was added to the medium for 24 h.

To evaluate whether TUG1 regulates PD-L1 expression, TUG1 was suppressed in two HCC cell lines (Fig. 3A). qRT-PCR showed that after TUG1 expression was downregulated, the mRNA expression of PD-L1 was also decreased (Fig. 3B). Moreover, PD-L1 protein levels were also significantly downregulated in shTUG1 cells compared with control cells (Fig. 3C). In addition, we found that IFN-γ could upregulate PD-L1 protein and mRNA expression (Fig. 3D; Supplemental Fig. 1A). More importantly, IFN-γ treatment restored the protein and mRNA expression of PD-L1 in the shTUG1 group (Fig. 3E; Supplemental Fig. 1B).

Next, we evaluated whether TUG1 inhibition affects HCC cells by allowing them to escape T cell-mediated killing. We cocultured effector T cells with HCC cells in vitro and evaluated the HCC cells with a CCK-8 assay. Indeed, knocking down TUG1 expression enhanced the T cell-mediated killing of MHCC-97H and HCC-LM3 cells (Fig. 3F). Together, these results suggested that silencing TUG1 could decrease PD-L1 mRNA and protein expression and inhibit HCC cells escape from T cell-mediated killing.

### TUG1 may inhibit PD-L1 through the JAK2/STAT3 pathway

**Figure 4:**
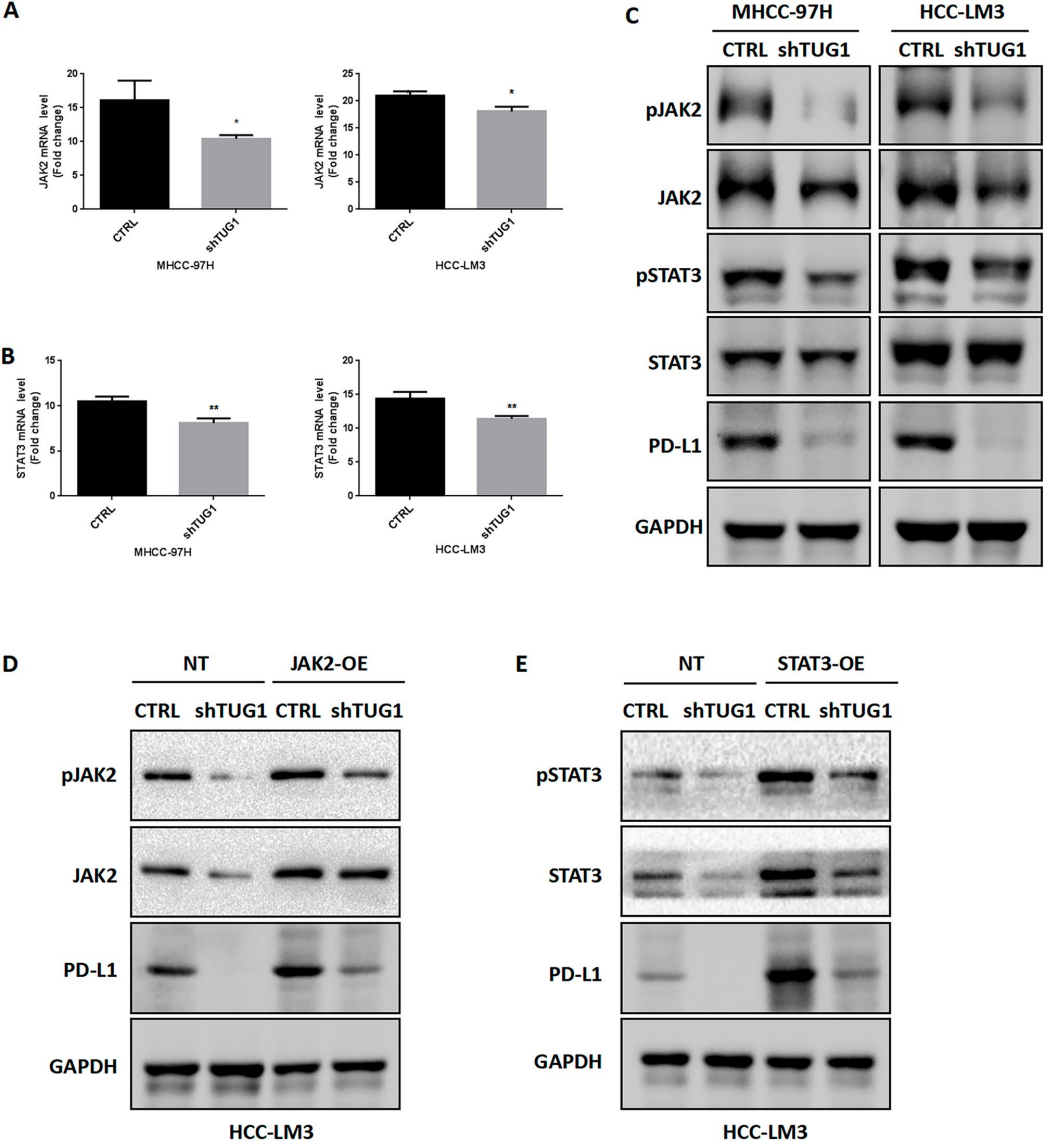
TUG1 affects the JAK2/ STAT3 pathway to regulate PD-L1. (**A**): The relative mRNA expression of JAK2 was analyzed in shTUG1 HCC cells. (**B**): The relative mRNA expression of STAT3 was analyzed in shTUG1 HCC cells. (**C**): The protein levels of pJAK2, JAK2, pSTAT3, STAT3, PD-L1 were analyzed in shTUG1 HCC cells by western blotting. (**D**) pJAK2, JAK2,and PDL1 protein levels are assessed by immunoblotting in HCC-LM3 cell was transfected with TUG1 silenced (shTUG1) and/or JAK2 overexpressed (JAK2-OE). (**E**) pSTAT3,STAT3,and PDL1 protein levels are assessed by immunoblotting in HCC-LM3 cell was transfected with TUG1 silenced (shTUG1) and/or STAT3 overexpressed (STAT3-OE).

Next, we investigated the mechanisms by which TUG1 decreases PD-L1 expression in HCC cells. The JAK2/STAT3 pathway is considered to play a significant role in regulating PD-L1^28^. Therefore, we speculated that TUG1-mediated inhibition of PD-L1 might occur through the JAK2/STAT3 pathway. We first detected JAK2 and STAT3 mRNA and protein levels in shTUG1 and control HCC cell lines. These results showed that JAK2 and STAT3 mRNA expression was decreased in the shTUG1 group compared with the control group (Fig. 4A,B). Similarly, silencing TUG1 significantly reduced phosphorylation level of JAK2 and STAT3 in HCC cells (Fig. 4C). Therefore, these results indicate that TUG1 can positively regulate the expression of JAK2 and STAT3.

To further establish whether JAK2 and STAT3 can modulate PD-L1 expression in HCC, we treated cells with AZ960 (JAK2 inhibitor) and A12232 (STAT3 inhibitor). As expected, after 24 h, AZ960 and A12232 treatment of HCC cells caused significant decreases in PD-L1 mRNA and protein expression compared with no treatment (Supplemental Fig. 2A, B).In addition, we treated the HCC-LM3 cell with shTUG1 and/or JAK2/STAT3 overexpression, and evaluated the levels of PDL1. As shown in Fig. 4D,E, JAK2 or STAT3 overexpression can abrogate down-regulation of PDL1 caused by the silencing of TUG1 expression. Overall, these results support a potential mechanistic role for TUG1-induced reduction in PD-L1 expression mediated via the JAK2/STAT3 pathway.

## Discussion

LncRNAs participate in the processes of various cancers in various roles. TUG1 is closely related to invasion, metastasis, proliferation and apoptosis in a variety of tumors, but its specific mechanism of action in hepatocellular carcinoma requires further exploration^29,30^. Lin et al.^31^ reported that TUG1 expression is downregulated in NSCLC and correlates with tumor differentiation grade. However, TUG1 expression was found to be upregulated in HCC tissues, and the expression of TUG1 was significantly positively correlated with tumor size and liver cancer stage^32^. Therefore, the role of TUG1 in cancer progression is still controversial. In this study, we first evaluated TUG1 expression in various cancers and its correlation with patient prognosis. Furthermore, we confirmed the results in HCC tumor and paired normal tissue samples by using RT-PCR to measure TUG1 mRNA expression. These results indicated that high TUG1 expression in HCC indicated a poor prognosis in HCC patients. These results were similar to those of previous studies^18,32^.

Recently, an increasing number of studies have shown that long noncoding RNAs play significant roles in immune regulation^33^. Xu et al.^34^ found that lncSros1 blocked the binding of *Stat1* mRNA to the RBP CAPRIN1 and promoted IFN-γ–STAT1-mediated innate immunity. Li et al.^22^ integrated multiomic data for 33 cancer types and identified several lncRNAs related to immune pathways. They also found that these immune lncRNAs are significantly correlated with immune cell infiltration. In hepatocellular carcinoma, Jiang et al.^35^ reported that lncRNA-EGFR stimulates Treg differentiation, suppresses CTL activity and promotes HCC growth in an EGFR-dependent manner. In the current study, we found that LncTUG1 expression is closely associated with CD4 T cells and macrophages in HCC.

With in-depth study of the tumor microenvironment, researchers have discovered that the tumor microenvironment can help tumors escape attack by the human immune system^36,37^. Activation of the PD-1/PD-L1 signaling pathway is supports tumor escape from immune surveillance, but the specific tumor immune escape mechanism is not yet clear^38–40^. PD-L1 has been shown to be closely related to tumor immune escape in a variety of tumors. For example, in non-small lung cancer, PD-L1 expression can help tumor cells escape attack by autoimmune cells, thereby affecting the prognosis of patients^12,41^. Innate immune cells like tumor-associated macrophages (TAMs), neutrophils (TANs), and dendritic cells mainly exert the role of promoting tumor progression. Research confirmed that high TUG1 expression and high levels of infiltration of pro-tumor immunocytes in liver cancer tissue leading to the malignant progression^20^. Study also found that lnc-TUG1 was significantly correlated with quiescent NK cells, suggesting that lnc-TUG1 had the potential to become a tumor immunotherapy target^19^. We identified that the expression of TUG1 was positively correlated with PD-L1 expression in HCC. Knockdown of TUG1 expression could reduce the mRNA and protein expression of PD-L1. Furthermore, our results showed that silencing TUG1 expression could partially improve effector T cell-induced killing of cancer cells. These results indicate that TUG1 can decrease PD-L1 expression in tumor cells, which subsequently affects anticancer immunity.

Current studies indicate that activation of the JAK/STAT pathway promotes the occurrence and development of various diseases, including various inflammatory diseases, lymphoma, leukemia, and solid tumors^42–44^. Moreover, there is mounting evidence that JAK2/STAT3 signaling regulates PD-L1 in many cell types^45,46^. Guru et al.^47^ found that the V617F mutation in JAK2 is accompanied by increased PD-L1 expression and that this PD-L1 overexpression is mediated by JAK2 (V617F) mainly through STAT3. miR-375 overexpression suppresses PD-L1 expression in gastric cancer via the JAK2/STAT3 signaling pathway^48^. In this study, we observed that silencing TUG1 decreased JAK2/STAT3 expression and that inhibition of JAK2/STAT3 signaling reduced the expression of PD-L1. Thus, our results indicate that TUG1 positively regulates PD-L1 via the JAK2/STAT3 signaling pathway and can be considered a promising therapeutic target in HCC.

## Conclusion

In summary, our study demonstrates that TUG1 and PD-L1 are highly expressed in liver cancer tissues and have potential as biomarkers and therapeutic targets. Importantly, we discovered that silencing TUG1 expression could improve the effector T cell killing capacity by suppressing the JAK2/STAT3/PD-L1 signaling pathway in vitro, which suggests that LncTUG1 may be developed to improve PD-1/PD-L1-based therapeutics for HCC. There are limitations to this study that need to be acknowledged. Firstly, additional hepatocellular carcinoma and adjacent normal tissue samples are needed to validate the results obtained at protein level and RNA level and to analyze clinicopathological relationships. Secondly, more comprehensive in vivo and in vitro studies are necessary to elucidate the mechanisms of TUG1 promotes hepatocellular carcinoma immune evasion via upregulating the JAK2/STAT3/PD-L1 signaling pathway. Lastly, clinical translation needs to be investigated to optimize therapeutic application. These work are the focus of our later research.

### Supplementary Information

Supplementary Legends.

Supplementary Figure 1.

Supplementary Figure 2.

Supplementary Table 1.

## Data Availability

The datasets used and/or analyzed in the current study are available from the corresponding author upon request.

## Abbreviations

TUG1: Taurine upregulated gene 1
HCC: Hepatocellular carcinoma
PD-L1: Programmed cell death ligand-1
qRT-PCR: Quantitative real-time polymerase chain reaction
