# Supplementary material for: LncTUG1 promotes hepatocellular carcinoma immune evasion via upregulating PD-L1 expression
Source: Sci Rep. 2023 Oct 9;13:16998. doi: 10.1038/s41598-023-42948-8 (PMC10562488; doi:10.1038/s41598-023-42948-8)
Supplement: Supplementary file 1 — Supplementary Legends. [file 41598_2023_42948_MOESM1_ESM.pdf]

Supplement Fig 1: IFN-γ stimulation upregulated the mRNA expression of PD-L1.

A: The relative mRNA expression of PD-L1 was increased under IFN-γ stimulation.

B: IFN-γ stimulation restored PD-L1 mRNA expression in shTUG1 HCC cells.

Supplement Fig2: Inhibition of JAK2/STAT3 suppresses the mRNA and protein expression of PD-L1

A: The relative mRNA expression of PD-L1 was reduced in HCC cells treated with AZ960 and A12232.

B: The protein levels of PD-L1 were reduced in HCC cells treated with AZ960 and A12232. To treat HCC lines, AZ960 (10 μmol/ml, Adooq Bioscience), A12232 (10 μmol/ml, Adooq Bioscience) ,were added to the medium for 24 h.
