# Supplementary figures and images for: LncTUG1 promotes hepatocellular carcinoma immune evasion via upregulating PD-L1 expression
Source: Sci Rep. 2023 Oct 9;13:16998. doi: 10.1038/s41598-023-42948-8 (PMC10562488; doi:10.1038/s41598-023-42948-8)

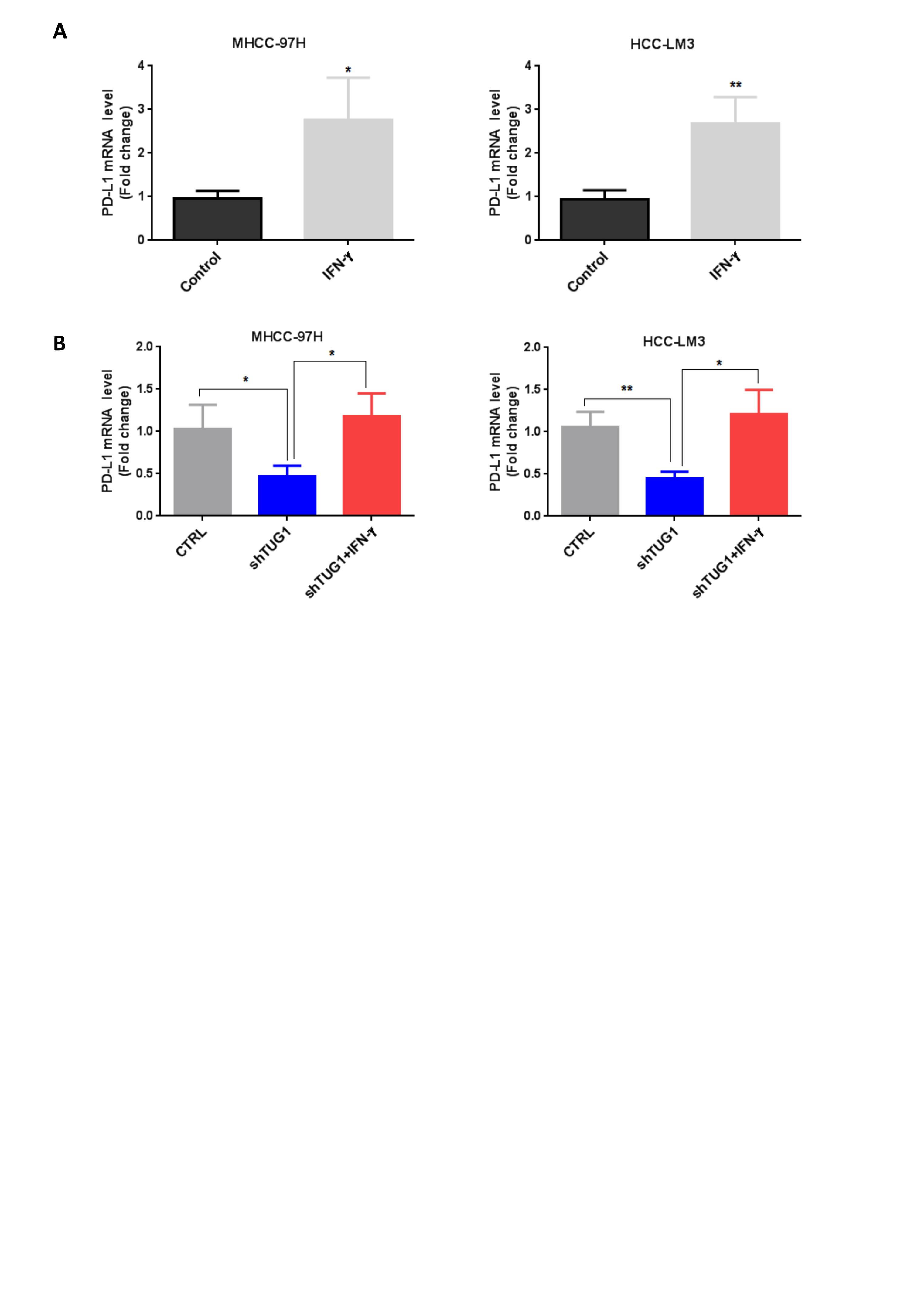

Supplement: Supplementary file 2 — Supplementary Figure 1. [file 41598_2023_42948_MOESM2_ESM.tiff]

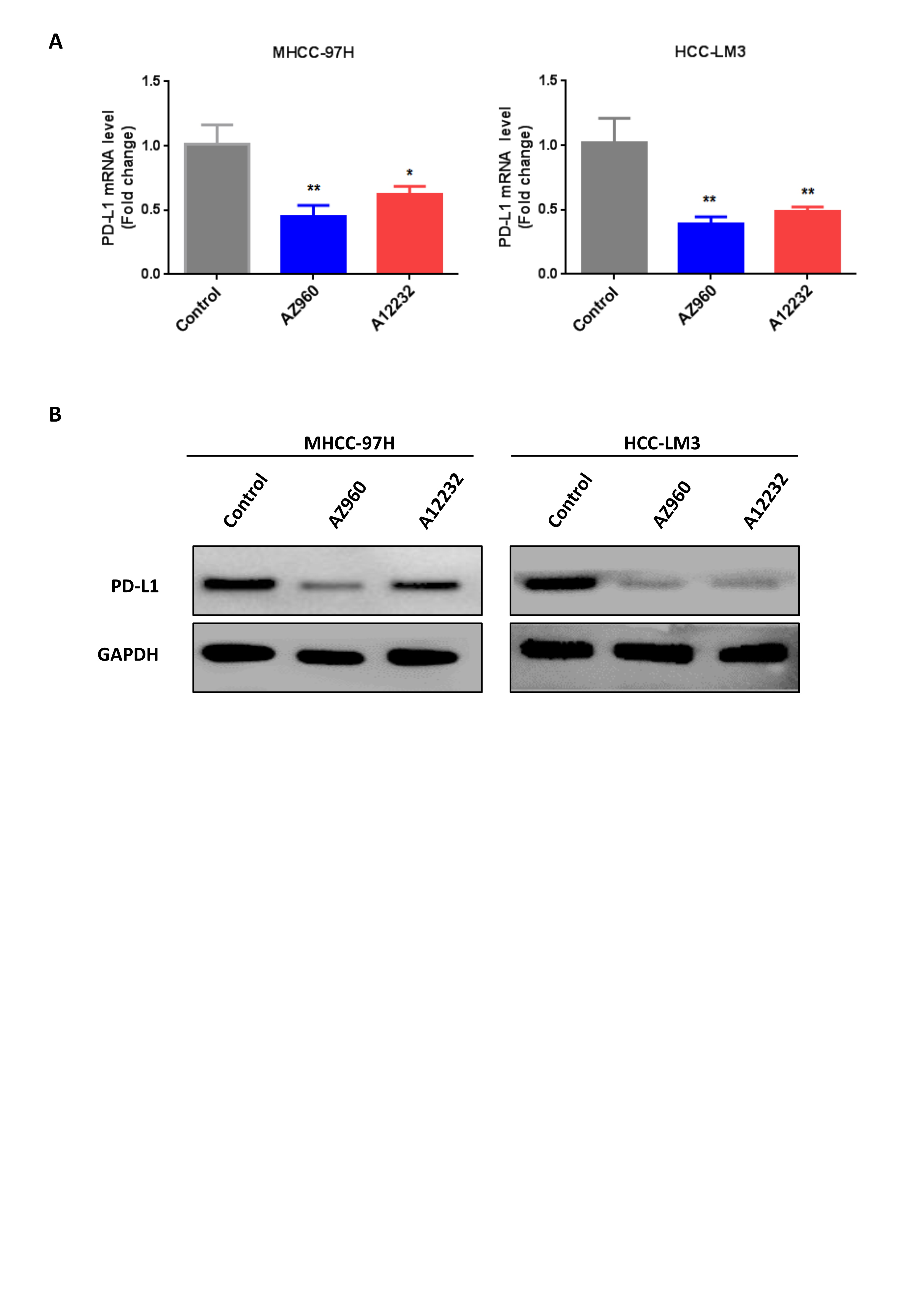

Supplement: Supplementary file 3 — Supplementary Figure 2. [file 41598_2023_42948_MOESM3_ESM.tiff]
