# Supplementary material for: LncTUG1 promotes hepatocellular carcinoma immune evasion via upregulating PD-L1 expression
Source: Sci Rep. 2023 Oct 9;13:16998. doi: 10.1038/s41598-023-42948-8 (PMC10562488; doi:10.1038/s41598-023-42948-8)
Supplement: Supplementary file 4 — Supplementary Table 1. [file 41598_2023_42948_MOESM4_ESM.docx]

Supplement Table 1: The primer pairs were used for human target gene in the study

| **Target Gene** | **Primer(5’-3’)** |
| --- | --- |
| TUG1 | Forward:GACTCCTTGAAAGCAGGGTCC  Reverse:CTCCTCCTTCCTAATAAATGCATCT |
| PD-L1 | Forward:TTGCTGAACGCCCCATACAA  Reverse:TCCAGATGACTTCGGCCTTG |
| STAT3 | Forward:CGGCGTCCAGTTCACTAC  Reverse:CCCAGAGTCTTTGTCAATGC |
| JAK2 | Forward:TTGCCAAAGGACATTCTTCAG  Reverse:AGAATCCAGAGCACTTAGAGG |
| GAPDH | Forward:GGAGCGAGATCCCTCCAAAAT Reverse:GGCTGTTGTCATACTTCTCATGG |
